# Characterization of Kbot21 Reveals Novel Side Chain Interactions of Scorpion Toxins Inhibiting Voltage-Gated Potassium Channels

**DOI:** 10.1371/journal.pone.0137611

**Published:** 2015-09-23

**Authors:** Rym ElFessi-Magouri, Steve Peigneur, Houcemeddine Othman, Najet Srairi-Abid, Mohamed ElAyeb, Jan Tytgat, Riadh Kharrat

**Affiliations:** 1 Laboratoire des Venins et Molécules Thérapeutiques, Institut Pasteur de Tunis,13 Place Pasteur, BP-74, 1002, Tunis, Tunisie; 2 Laboratory of Toxicology & Pharmacology, University of Leuven (K.U. Leuven), Campus Gasthuisberg O&N2, Herestraat 49, P.O. Box 922, B-3000, Leuven, Belgium; Indiana University School of Medicine, UNITED STATES

## Abstract

Scorpion toxins are important pharmacological tools for probing the physiological roles of ion channels which are involved in many physiological processes and as such have significant therapeutic potential. The discovery of new scorpion toxins with different specificities and affinities is needed to further characterize the physiology of ion channels. In this regard, a new short polypeptide called Kbot21 has been purified to homogeneity from the venom of *Buthus occitanus tunetanus* scorpion. Kbot21 is structurally related to BmBKTx1 from the venom of the Asian scorpion *Buthus martensii* Karsch. These two toxins differ by only two residues at position 13 (R /V) and 24 (D/N).Despite their very similar sequences, Kbot21 and BmBKTx1 differ in their electrophysiological activities. Kbot21 targets K_V_ channel subtypes whereas BmBKTx1 is active on both big conductance (BK) and small conductance (SK) Ca^2+^-activated K^+^ channel subtypes, but has no effects on Kv channel subtypes. The docking model of Kbot21 with the Kv1.2 channel shows that the D24 and R13 side-chain of Kbot21 are critical for its interaction with K_V_ channels.

## Introduction

Scorpion venom is a source of interesting bioactive compounds, such as neurotoxins which are invaluable tools for studying structure and function of potassium channels [1] and are now serving as templates for the development of molecular therapeutics [2,3].

The subtypes of K^+^ channels targeted by scorpion toxins include voltage-gated [4], inward rectifier [5], ether-a-go-go-related gene [6–8] and Ca^2+^-activated channels including large, intermediate and small conductance channels [9–11]. These channels play a key role in the regulation of a wide variety of physiological processes involved in cell excitability, such as regulation of muscle contraction,heartbeat, hormonal secretion, signal transduction, neurotransmitter release, and cell proliferation [12–14].

These toxins (KScTxs) are short-chain peptides of 28 to 40 amino acids, with three or four disulfide bridges. Their structures exhibit a common minimal motif, named the “Cystein-Stabilized-Helix” (CSH) [15, 16]. These toxins have been extensively investigated and mutation studies have identified critical residues important for both structural and functional properties. Dauplais et al. [17] have demonstrated that lysine at position 27 in charybdotoxin from *Leiurus quinquestriatus hebraeus* physically occludes the pore of K_V_1 channels, thus preventing the flow of K^+^ ions. This lysine and an aromatic residue (tyrosine or phenylalanine) separated by 6.6±1.0 Å forms the functional dyad which is essential to target K_V_ channels [17]. Furthermore, mutagenesis of charybdotoxin highlighted several positions which are important for its binding on K_V_ channels and large conductance Ca^2+^ activated channels (BK). These include residues S10, W14, R25, M29 and R34 where mutations led to drastic reduction in binding affinity. The functional residues for K_V_ and BK channels are located on the β-sheets in contrast to these of ERG and SKCa channels which are located at the α-helix [9].

Despite the fact that there is a homologous structural folding and similar architecture of the vestibule of the channel pore, binding of scorpion toxins is characteristic for each type of K^+^ channel. Thus, channels and/or toxins should have subtle differences that would explain the specific interactions found for each channel–toxin pair. For example, Iberiotoxin that is highly specific for BK channel has G30 instead of N30 (a residue maintained for most scorpion toxins), and the mutation of this glycine to asparagines allowed the mutant Iberiotoxin [G30N] to target both K^+^ channel subtypes [11, 18–19].

In this paper, we have described the biochemical and functional characterization of Kbot21 isolated from the venom of *Buthus occitanus tunetanus*. Furthermore, using the two electrode voltage clamp technique combined with docking models we investigated the structure-function relationship of this toxin.

## Materials and Methods

Authors attest that experiment on animals were carried out in accordance with the European Community Council Directive (86/609/EEC) for experimental animal care and all procedures met with the approval of the Institutional Research Board of the Pasteur Institute of Tunis. the ethics approval did not cover the experiments on the scorpion because they are invertebrates. The use of the frogs was in accordance with the license number LA1210239 with the approval of Laboratory of Toxicology & Pharmacology, University of Leuven.

### Scorpion venom

*Buthus occitanus tunetanus* venom was provided in liquid state by electric stimulation of the post-abdomen of the scorpion, bred in captivity in Beni Khedach area (Tunisia). The pooled venom is kept frozen at –20°C in its crude form until use. All reagents were purchased from Sigma Aldrich® chemical company, except indicated otherwise.

### Purification of Kbot21

Purified Kbot21 was obtained from the scorpion venom *Buthus occitanus tunetanus* by gel filtration G50 followed by HPLC.

Crude venom was dissolved in water and loaded on to a sephadex G50 column equilibrated with 0.1M ammonium acetate pH 8.5. Different fractions were eluted and tested for their toxicity on mice. Only fraction (BotG50) showing a toxic activity [20–21] was then applied onto C8 semi preparative reversed-phase HPLC column (10 mm x 250mm, 5 μm, Beckman Fullerton) equilibrated in 0.1% trifluoroacetic acid in water, at a flow rate of 1ml/min. HPLC purification of the non-retained fraction was performed using an analytical C18 reversed-phase HPLC column (4.6mm x 250 mm, 5 microns Beckman). Elution was monitored at 214nm.

### Molecular weight determination

Molecular weight of Kbot21 was first estimated by SDS-PAGE analysis under non-reducing conditions with a stacking gel of 3% (w/v) (pH 6.8) and a running gel of 15% (w/v) (pH 8.8). Both types of gel were fixed and stained with silver nitrate and dried under vacuum.

Then, the peptide was then analyzed on a voyager de RP MALDI-TOF mass spectrometer (Perspective Biosystems, Inc., Framingham, MA). Sample was dissolved in CH3CN/H2O (30/70) with 0.3% trifluoroacetic acid to obtain a concentration of 1–10 pmol.μl^-1^. The matrix was prepared as follows: alpha–cyanohydroxycinnamic acid was dissolved in 50% CH3CN in 0.3% trifluoroacetic acid/H2O to obtain a saturated solution at 10 μg.μl^-1^. A 0.5 μl of peptide solution was placed on the sample plate, and 0.5μl of the matrix solution was added. This mixture was allowed to dry. Mass spectra were recorded in linear mode, were externally calibrated with suitable standards and was analysed by using the GRAMS/386 software.

### Amino acid sequence determination

Reduction and alkylation of proteins, and sequence determination of native and S-alkylated peptides were performed as described in [22].

The automatic Edman degradation of 1 nmol of native Kbot21 was performed with a reproducible yield of 95% during the first 31 cycles. After these steps, the concentration of identified PTH became too low for reliable identification. Then, 1nmol of S-alkylated-protein was used to complete the sequence determination of Kbot21 and to identify cystein positions.

### Toxicity test

Kbot21 was tested for in *vivo* toxicity on 20 ± 2 g male C57/ Bl/6 mice, by intracerebro-ventricular injection (i.c.v), considered as the most sensitive route to mammals for scorpion toxins. Protein was diluted in 0.1% (w/v) BSA and 5μl of the solution containing increasing amounts of peptides were injected in six mice for each concentration [23–24]. Our investigation of toxins was performed by persons with appropriate training. Experiments on mice were carried out in accordance with the European Community Council Directive (86/609/EEC) for experimental animal care, and all procedures met with the approval of the Institutional Research Board of the Pasteur Institute of Tunis.

In our experiments the death as an end-point cannot be avoided, but the experiments are designed to result in the deaths of as few animals as possible. We are also ensuring that the animal's suffering or pain is minimized.

When it is necessary to kill animal, humane procedures was used. These procedures avoid distress, be reliable and produce rapid loss of consciousness without pain until death occurs. Ether was used during this study as an anesthetic or to humanely sacrifice the mice.

### Expression in *Xenopus* oocytes

For the expression of the VGPCs (rK_V_1.1, rK_V_1.2, hK_V_1.3, rK_V_1.4, rK_V_1.6, *Shaker* IR, rK_V_2.1) in *Xenopus* oocytes, the linearized plasmids were transcribed using the T7 or SP6 mMESSAGE-mMACHINE transcription kit (Ambion, USA). The harvesting of stage V-VI oocytes from anaesthetized female *Xenopus laevis* frog was previously described [25]. Oocytes were injected with 50 nl of cRNA at a concentration of 1 ng/nl using a micro-injector (Drummond Scientific, USA). The oocytes were incubated in ND96 solution containing (in mM): NaCl, 96; KCl, 2; CaCl_2_, 1.8; MgCl_2_, 2 and HEPES, 5 (pH 7.4), supplemented with 50 mg/l gentamycin sulfate. The use of the frogs was in accordance with the license number LA1210239.

### Electrophysiological recordings

Two-electrode voltage-clamp recordings were performed at room temperature (18–22°C) using a Geneclamp 500 amplifier (Molecular Devices, USA) controlled by a pClamp data acquisition system (Axon Instruments, USA). Whole cell currents from oocytes were recorded 1–4 days after injection. Bath solution composition was ND96 (in mM): NaCl, 96; KCl, 2; CaCl_2_, 1.8; MgCl_2_, 2 and HEPES, 5 (pH 7.4). Voltage and current electrodes were filled with 3 M KCl. Resistances of both electrodes were kept between 0.7–1.5 MΩ. The elicited currents were filtered at 1 kHz and sampled at 500 Hz using a four-pole low-pass Bessel filter. Leak subtraction was performed using a-P/4 protocol. K_V_1.1-K_V_1.6 and *Shaker* IR currents were evoked by 500 ms depolarizations to 0 mV followed by a 500 ms pulse to -50 mV, from a holding potential of -90 mV. In order to investigate the current-voltage relationship, current traces were evoked by 10 mV depolarization steps from a holding potential of -90 mV. All data represent at least 3 independent experiments (n ≥ 3) and are presented as mean ± standard error.

### Molecular modelling and Protein-Protein Docking

#### Three-dimensional structure models of Kbot21 toxin and Kv1.2, Kv1.4 and Kv2.1 channels

The homology model of Kbot21 was built using the NMR structures of the BmBKTx1 peptide [26] (PDB code: 1Q2K). In order to partially account for the flexibility of the scorpion peptides, all the 21 NMR solutions presented in the PDB file were used as templates to build 21 models of Kbot21. The structural models were generated by simply mutating the 13^th^ (Val to Arg) and 24^th^ (Asn to Asp) amino acids using SCWRL4 program [27] followed by 50 and 500 steps of energy minimization using steepest-descent and adopted basis Newton-Raphson methods respectively.

The Kv1.2 is a potassium channel belonging to the Shaker-like subfamily. Its full length crystallography structure was solved for the first time by Long et al. [28]. However, in our study we used the crystal structure of Kv1.2-Kv2.1 paddle chimera channel due to its better quality [29]. In our study we were restricted only to the pore domain.

The homology models of Kv2.1 and Kv1.4 were build with MODELLER 9v8 [30] using only the pore domain of the channel crystal structure (PDB code: 2R9R) [29]. Sequences of human Kv2.1 and Kv1.4 were retrieved from Uniprot database (http://www.uniprot.org/) under Q14721 and P22459 codes, respectively. Twenty structures were generated for each subtype and their respective DOPE [31] scores were calculated. The models of Kv1.4 and Kv2.1 with the lowest DOPE values were selected and retained.

### Protein-Protein Docking

Solutions of protein-protein docking between Kbot21 and the Kv1.2 potassium channel were generated using ZDOCK software [32]. The 21 models of Kbot21 generated were used to run an ensemble docking. Consequently, a total of 42000 poses was returned. A clustering analysis was carried in order to remove redundancy due to the highly geometrical similarity of some complexes. Cα Root Mean Square Deviations (RMSD) of the whole set of docking solutions were calculated. The poses belonging to the same cluster were grouped together based on a RMSD cut-off of 4 Å.

The most represented clusters (>10 members) were then processed for the refinement using molecular mechanics for only the centre of each cluster. The refinement consists of 50 steps of Steepest-descent energy minimization followed by 800 steps of adopted basis Newton-Raphson minimization. Both refinement stages were run while constraining the backbone atoms of the complex with a constant force of 20 and 10 Kcal/mol/ Å^2^, respectively for each stage. We then used a scoring function based on a single point energy computation of the Molecular Mechanics Poisson Boltzmann Surface Area (MM-PBSA) method. The entropy term was not considered in the study.

## Results

### Purification of Kbot21

**Fig 1:**
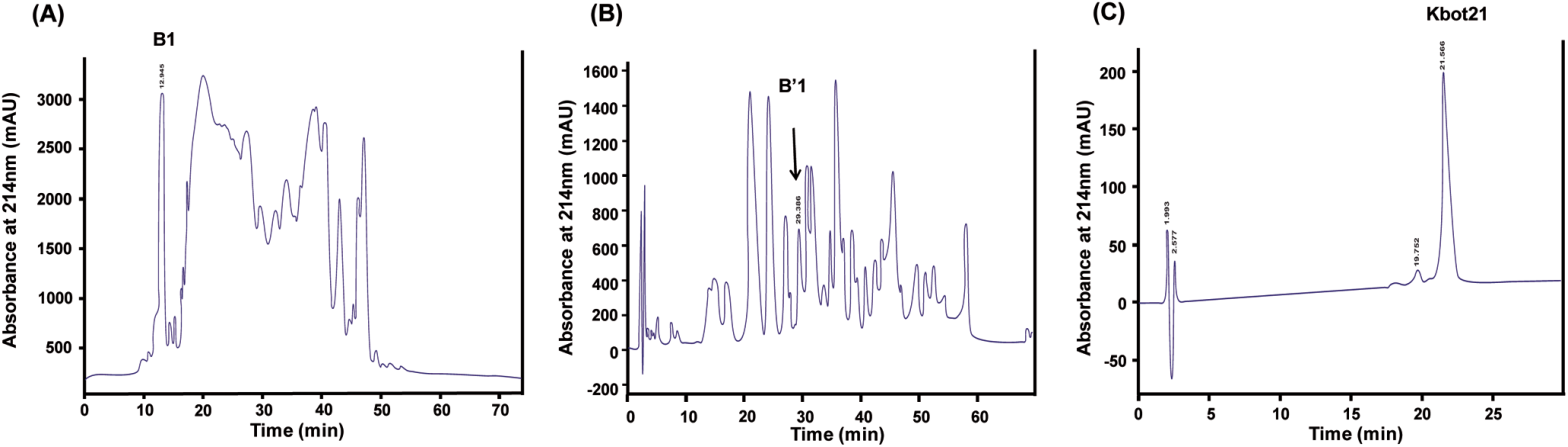
Purification of Kbot21 from scorpion *Buthus occitanus tunetanus* venom. **(A)** Chromatography of fraction BotG50 on semi preparative C8 reverse phase HPLC column. **(B)** Chromatography of fraction B1on C18-RP-HPLC, B’1 was collected at 29min. **(C)** Kbot21 was purified from the fraction B’1 by C18-RP-HPLC. It is collected at 21min.

The toxic fraction obtained from Sephadex G-50 chromatography of the *Buthus occitanus tunetanus* scorpion venom was fractionated by a C8 semi preparative reversed-phase HPLC (Fig 1A). Fraction B1 containing peptides in the range of 3.7–4.6 KDa, according to SDS-PAGE, were collected, lyophilised and loaded on to an analytical C18 reverse-phase HPLC column (Beckman Fullerton) using a linear gradient (12% to 35% in 70 min). The fraction B’1 was composed of three fractions when eluted by more resolutive gradient (12 to 30% in 30 min) (Fig 1B). The most representative fraction shows only one band at about 4 kDa on SDS-PAGE electrophoresis and a single component with molecular weight of Da by mass spectrometry obtained by MALDI is nearly identical with the average theoretical molecular mass calculated for the fully oxidized form of Kbot21 (Da) (data not shown). This molecule, named Kbot21 (Fig 1C).

### Kbot21 exhibit Acute Toxicity following i.c.v injection to mice

We injected increasing doses, from 0.8 to 25 ng, of Kbot21 to two sets of C57bL/6 mice containing each six individuals by i.c.v. The LD50 value corresponds to 20 ng/g mice or 1μg/kg body weight which is 5 times to other LD50 values of some scorpion peptides, such as Maurotoxin, well characterized for their activity on potassium channels (LD50 = 100 ng) [33].

### Sequence determination and comparison with other scorpion toxins

**Fig 2:**
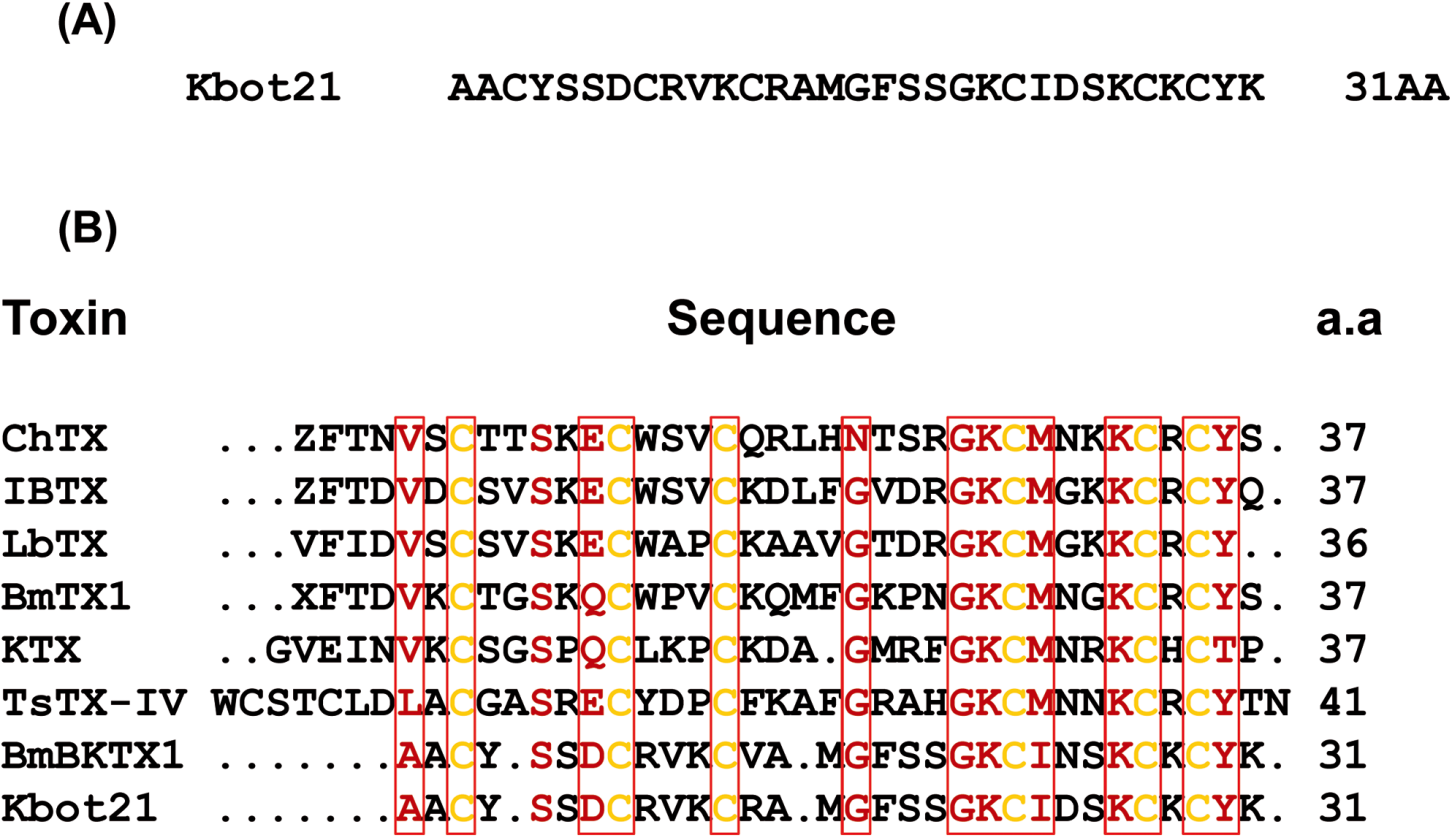
Consensus sequences and comparison with other α KTx scorpion toxins. **(A)** Amino acid sequence of reduced and S-alkylated Kbot21 Cysteine residues were identified as PTH-S-pyridylethylcysteine. **(B)** Sequence comparison of α KTx different scorpion toxins K^+^ channel inhibitors. On the basis of amino acid sequence, the sequences have been aligned with respect to the six cysteine residues.

Kbot21 is a basic polypeptide composed of 31 amino acids with six conserved cysteines and shares less than 40% sequence identity with the known alpha-KTx toxins; (Fig 2A). The experimental mass of Kbot21 value was in a perfect accordance with the sequence obtained.

The amino-acid sequence alignment of Kbot21 with those of short chain toxins (Fig 2B), showed that it has nearly the same similarity with toxins of the KTX group: 34.2% (subfamily 3) and the NTX group: 36.8% (subfamily 2) (classification proposed by Tytgat et al. [34]) but Kbot21 is structurally related to BmBKTx1 (subfamily 19) from the venom of the Asian scorpion *Buthus martensi Karsch*. These two toxins differ by only two residues at position 13 (R /V) and 24 (D/N). Kbot21 could be considered as the 2nd member of the α-KTx19 subfamily (systematic number: α –KTx19.2). The protein sequence data reported in this paper will appear in the UniProt Knowledgebase under the accessionnumber C0HJQ2.

### Kbot21 inhibits Kv channels

**Fig 3:**
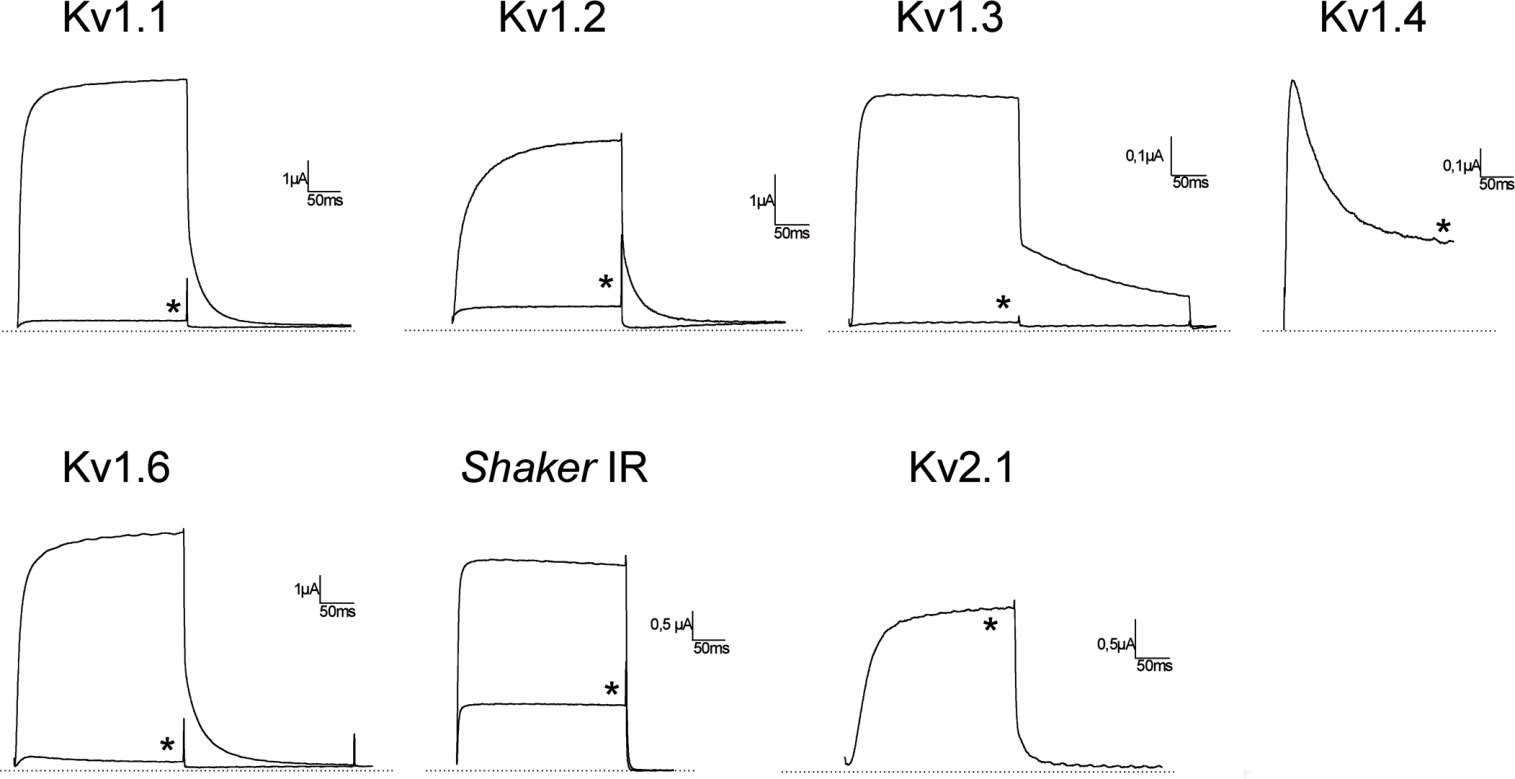
Electrophysiological activities of Kbot21. Screening on different K+ channel subtypes: The currents were measured in *X*. *laevis* oocytes heterologously expressing a single type of cloned voltage-gated potassium channel isoforms. Traces shown are representative of at least three independent experiments (n ≥ 3). The dotted line indicates the zero-current level. The asterisk (*) distinguishes the steady-state current after application of 1 μM of Kbot21.

Kbot21 was tested for its activity against 7 voltage-gated potassium channel isoforms. At a concentration of 1μM, Kbot21 was found to completely inhibited the potassium current of the mammalian Kv1.1, Kv1.2, Kv1.3 and Kv1.6 potassium channel subtypes. Furthermore, it inhibits 65% of the insect channel *Shaker* IR current. However, Kbot21 failed to exert any activity against Kv1.4 and Kv2.1 channels (Fig 3).

**Fig 4:**
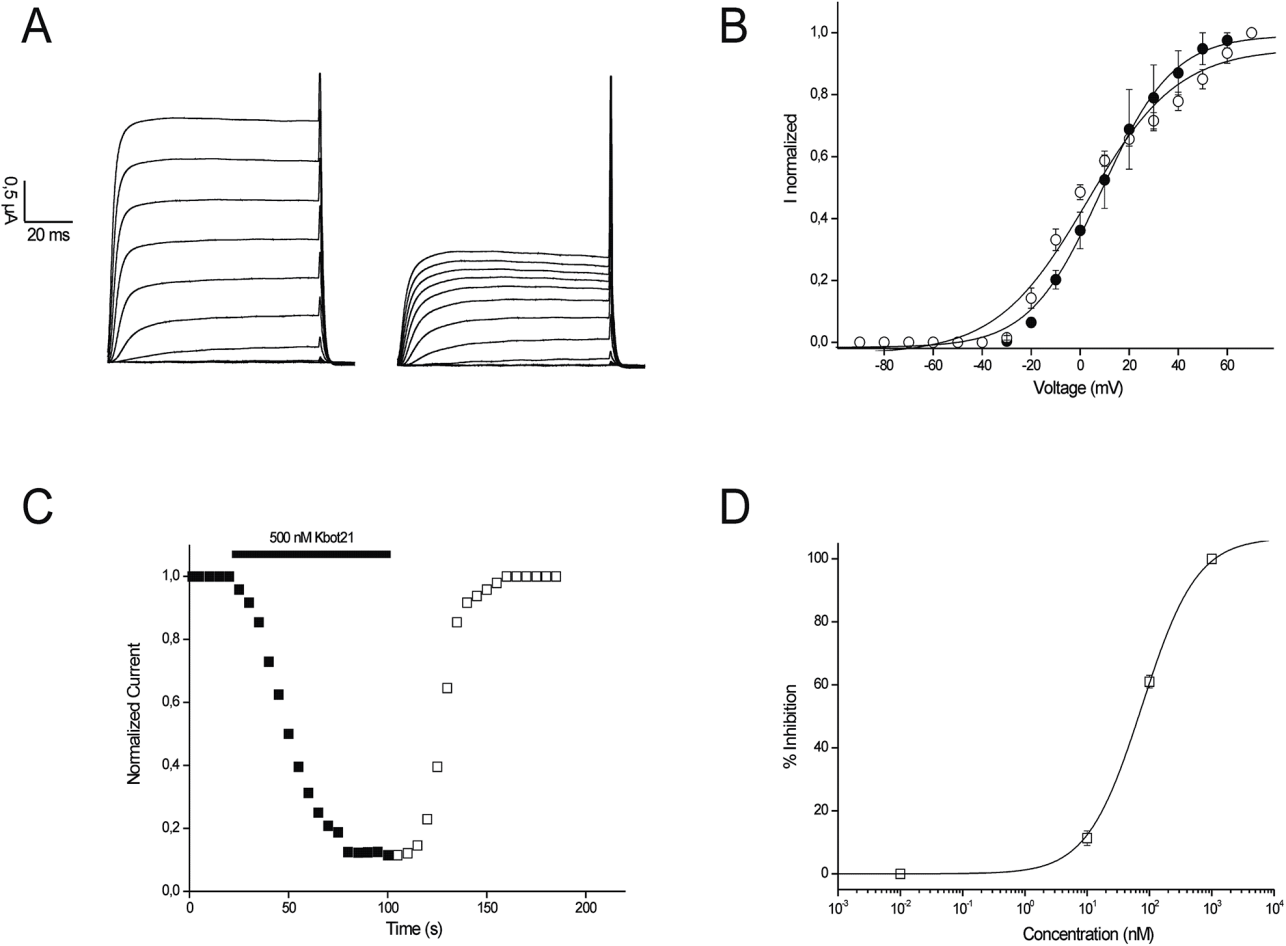
Electrophysiological charaterisation of Kbot21. **(A)** Current traces at different voltages in control (left panel) and in the presence of 100 nM toxin (right panel). **(B)** The plot shows the voltage-current relationship in control conditions and after addition of 100 nM Kbot21. The potentials were tested in a range from -80 mV to +70 mV. Closed circles show the V1/2 in control and the open circles the addition of 100 nM Kbot21. **(C)** Representative experiment of the time course of Kv1.6 current inhibition with Kbot21 (500 nM) and the reversibility hereof. Control (closed squares); washin (closed squares + black bar); washout (open squares). Blockage occurred rapidly, and binding was reversible upon washout. **(D)**The plot shows the concentration-response curve for Kbot21 on Kv1.6 channels obtained by plotting the percentage blocked current as a function of increasing toxin concentrations. All data represent at least 3 independent experiments (n ≥ 3) and are presented as mean ± standard error.

Kv1.6 channel was used to further characterize the effect of Kbot21 on channel gating. Fig 4A shows current traces at different voltages in control and after application of 75 nM Kbot21. Kbot21 did not significantly shift the midpoint of activation. The V_1/2_ yielded 8.5 ± 0.9 mV in control and 6.9 ± 2.9 mV after application of 100 nM Kbot21 (n ≥ 3) (Fig 4B). Blockage of Kv1.6 channels occurred rapidly and its binding was reversible since the current recovered quickly and completely upon washout (n = 3) (Fig 4C). In order to assess the potency, a concentration response curve was constructed (Fig 4D). An IC_50_ value of 75.9 nM was found with a Hill coefient of H = 1.1 ± 0.1.

### Computational study

**Fig 5:**
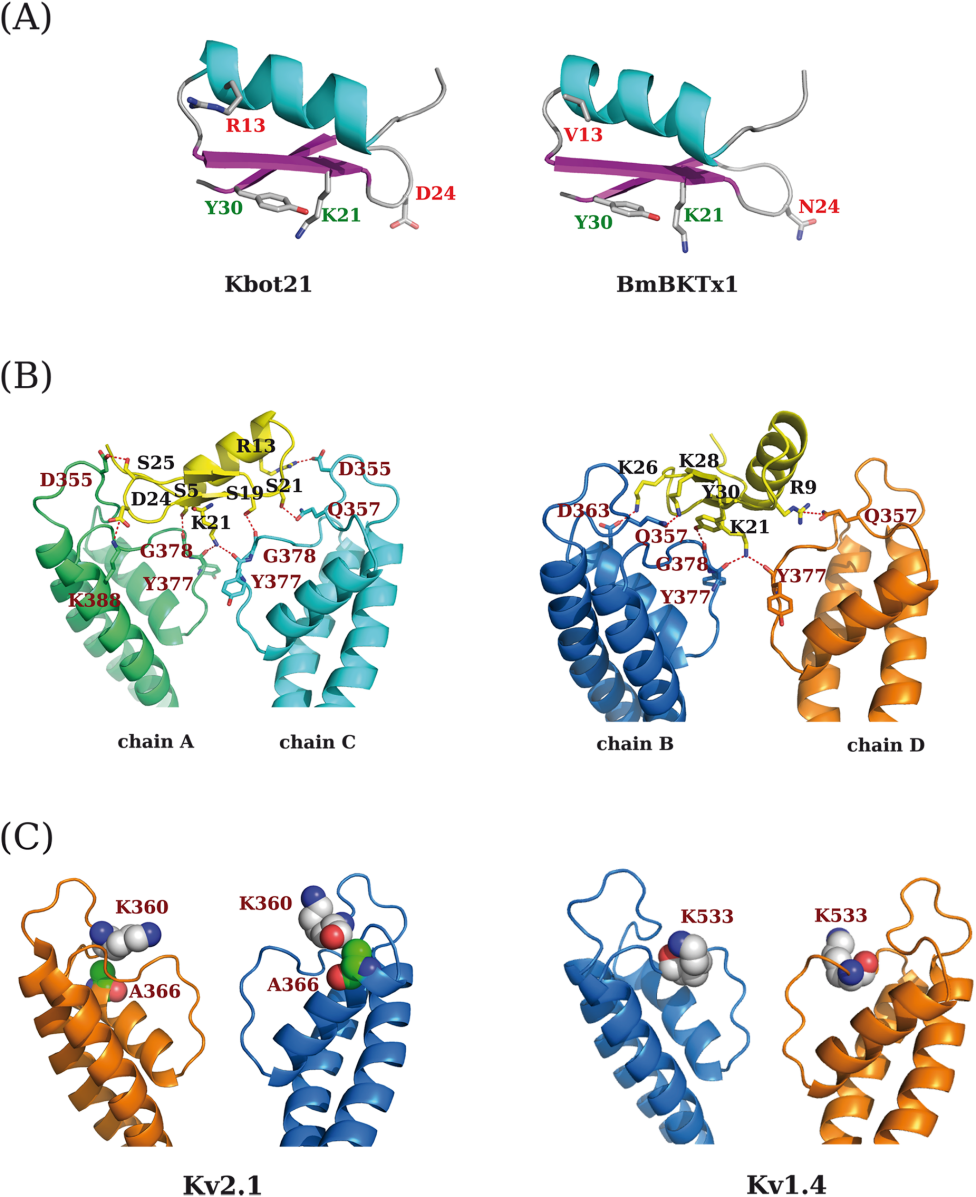
Molecular modelling. **(A)** the 3D model of Kbot21 generated from the NMR structure of BmBKTx1. The amino acids of the functional dyad, K21 and Y30, and the different residues between the two peptides at positions 13 and 24 are shown. **(B)** The interaction mode of Kbot21 on the Kv1.2 potassium channel as predicted from protein-protein docking study. **(C)** The Kv2.1 and Kv1.4 potassium channels subtype structure model. Only two opposed chains are represented for clarity. The residues K360 and A366 for Kv2.1 and K531 for Kv1.4 are suggested being responsible for the ineffectiveness of Kbot21 on these two potassium channel subtypes.

The best top solution of Kbot21-Kv1.2 channel docking describes a mechanism of blocking in which Lys21 interacts with the outer most carbonyl groups of the potassium channel selectivity filter. The hydroxyl of Y30 establishes a hydrogen bond with the carbonyl group of the G393 of Kv1.2 channel. The distance between K21 and Y30 of Kbot21 is about 6 Å. This mechanism highlights a functional dyad described by Dauplais et al. [17] (Fig 5A).

Furthermore, to hold tight the complex, the peptide establishes also many other interactions (Fig 5B), mainly of polar nature. Kbot21 presents three salt bridges by the mean of R13, K26 and D24 interacting with D355, D363 and K388 in Kv1.2 channel respectively. It is also noted that the D370 residue interacts with R369 in Kv1.2 via two hydrogen bonds, which in turn, interacts with R13 of Kbot21. In addition, K28 interacts with D394.

We studied the effect of mutating *in silico*, R13V13 / D24N24 or R13D14/V13N24 on the estimated binding energy of the complex (data not shown). The three mutants generated *in silico* present an increase of binding energy score comparable to that of Kbot21/Kv1.2 interaction. The most important one corresponds to the double mutation R13D24 / V13N24, which correspond to the sequence of BmBKTx1, where the score increases with +84 Kcal/mol. The binding energy for the D24/N24 mutant drastically increases with about 50 kcal/mol while for the R13/V13 mutation the value increases to nearly 20 Kcal/mol.

The Kv2.1-Kbot21 and Kv1.4-Kbot21 complexes were generated based on the Kv1.2-Kbot21 complex obtained by substuting the structure of Kv2.1 and Kv1.4 potassium channels subtypes (Fig 5C).

The Kv2.1 potassium channel has a K360 equivalent to Q372 in Kv1.2 which interacts with R9 and K28 of Kbot21. The distance between K360 of Kv2.1 and the ionizable groups of R9 and K28 of Kbot21 is of 6.5 Å and 6 Å respectively. In addition, an interaction between K26 (positively charged) of Kbot21 and D378 (negatively charged) of Kv1.2, is not maintained in the case of Kv2.1 because the residue D378 of Kv1.2 is replaced by an aliphatic amino acid, A366, in Kv2.1. Thus, the increasing charge of the Kbot21 interaction surface is probably responsible for its lack of interaction with Kv2.1.

For Kv1.4 potassium channel subtype, the analysis of the generated complex revealed that R9 and R28 of Kbot21 opposes a positively charged amino acid, K531, localized on different subunits chains near the extracellular pore opening, abriously generating a repulsive effect.

## Discussion

The present study reports the purification and characterization of a new α-KTx scorpion toxin. The *α*-KTx family is the largest of the KScTx families and is divided into at least 22 subfamilies, defined according to the sequence alignments of the toxins [34,35]. Most of these toxins are less than 38 residues and exist in small amounts in scorpion venoms. Such peptides have usually been identified by bioassay-guided screening methods of scorpion venoms. In this work the venom of the scorpion *Buthus occitanus Tunetanus* was purified using a combination of gel filtration chromatography, RP-HPLC separation, and interval of bioassays and molecular mass determination.

This approach led to the purification of Kbot21. This is a basic polypeptide composed of 31 amino acids with six conserved cysteines and shares less than 40% sequence identity with the known alpha-KTx toxins; however it is a closely related analog of BmBKTx1 from the venom of the Asian scorpion *Buthus martensi* Karsch in a structural point of view. Kbot21 and BmBKtx1 are differing by only two residues at position 13 (R/V) and 24 (D/N). Our data show that, despite their high sequence similarity, Kbot21 and BmBKTx1 differ in their biological activities. Indeed it has been reported that BmBKTx1 is active on both big conductance (BK) and small conductance (SK) Ca^2+^-activated K^+^ channels but has no effects on K_V_ channels [36], However in this work, we showed that Kbot21 is a potent blocker of Kv1.1, Kv1.2, Kv1.3, Kv1.6 and *Shaker* K^+^ channels without being active on a Kv1.4 and Kv2.1 channels. This confirms that the modification of a critical functional residue can markedly affect the activity of a peptide. Toxins’ affecting multiple ion channels as a result of subtle changes in sequences has become a well accepted idea. Indeed, few of these peptides are selective for a given K^+^ channel subtypes, for example, Charybdotoxin [19], Maurotoxin [37]. and several others block both voltage-gated and Ca^2+^-activated K^+^ channels. Some toxins escape this rule, for example Iberiotoxin that is a selective blocker of large-conductance Ca^2+^- activated K^+^ channel [38]. and P05 is specific to small-conductance Ca^2+^-activated K^+^ channel [39].

Pairwise alignment does not differentiate critical functional residues (e.g. an active site) from residues with no critical role [40]. However, it is clear that the residue at position 24 plays an important role in modulating activity and specificity of both Kbot21 and BmBKTx1. Indeed the occupation of this position by an aspartic is quite unique in the sequence of Kbot21. This position is otherwise occupied by a Glycine or Asparagine in a majority of already known toxins. It has been shown that only a single residue difference in the functional surface distinguishes K_V_ and BK channels, where asparagines (at position 30 for CHTX) is specific to K_V_ channels [41]. This was determined from analyses of multiple sequence alignment of scorpion toxins that target different channel subtypes and from mutagenesis data (Fig 2B). P05 targets only SKCa channels and mutation at positions 22–24 from IGV to MNG did result in recognition of K_V_ channels [39]. Our results show for the first time that Asparagine can be substituted by Aspartic acid in the sequence of Kbot21 without losing activity against K_V_ channels. This is in contrast to BmBKTx1 which contains an Asparagine at this position and is not active on K_V_ channel but however is a specific K channel toxin. This is against the results obtained by mutagenesis studies performed on the Iberiotoxin showing the importance of the glycine residue for high specific interaction with BK channel [38]. However, our results corroborate with the study of Schroeder et al. [18] where they mutated glycine to asparagine at position 30 in Iberiotoxin. All these results suggest that the occupation of the position 24 by an asparagine / glycine is not sufficient to determine the specificity of action on the BK/ K_V_ channels, and probably the presence of other residues is required. To try to answer to this question we launched a very thorough study of protein-protein docking with the Kbot21 toxin and Kv1.2 channel. This study shows that Kbot21 acts on Kv1.2 by blocking the pore via its functional dyad consisting of K21 and Y30. Even though theorical, the model proposed in this interaction study, even though it is only a model, does provides useful information since the obtained mechanism lines very well with the generally accepted mechanism in which α-KTx acting on different voltage-dependent potassium channel subtypes, including ChTx [17,42]. Kbot21 did not alter the kinetics of channel gating, suggesting that this toxin exhibits its potassium channel inhibition by physically obstructing the potassium ion pathway, which is a further support of the proposed interaction model.

The proposed interaction model of Kbot21 with K_V_1.2 suggests the presence of several other interactions occurring mainly between polar residues. The most important is that of Kbot21’s D24 with K403 of K_V_1.2 resulting from bringing to closeness two opposite charged amino acids. The in *silico*, binding energy analysis of the Kbot21 mutants, suggests that this interaction is more critical for the binding than the interaction between R13 and D370. However their combined effect, which corresponds to the sequence of BmBKTx1, is more important and highlights the importance of both residues in the interaction with Kv1.2. Thus our model successfully predicts that Kbot21 is more active than BmBKTx1 on Kv1.2. This is in accordance with our experimental data obtained in the electrophysiological experiments (Fig 4).

**Fig 6:**
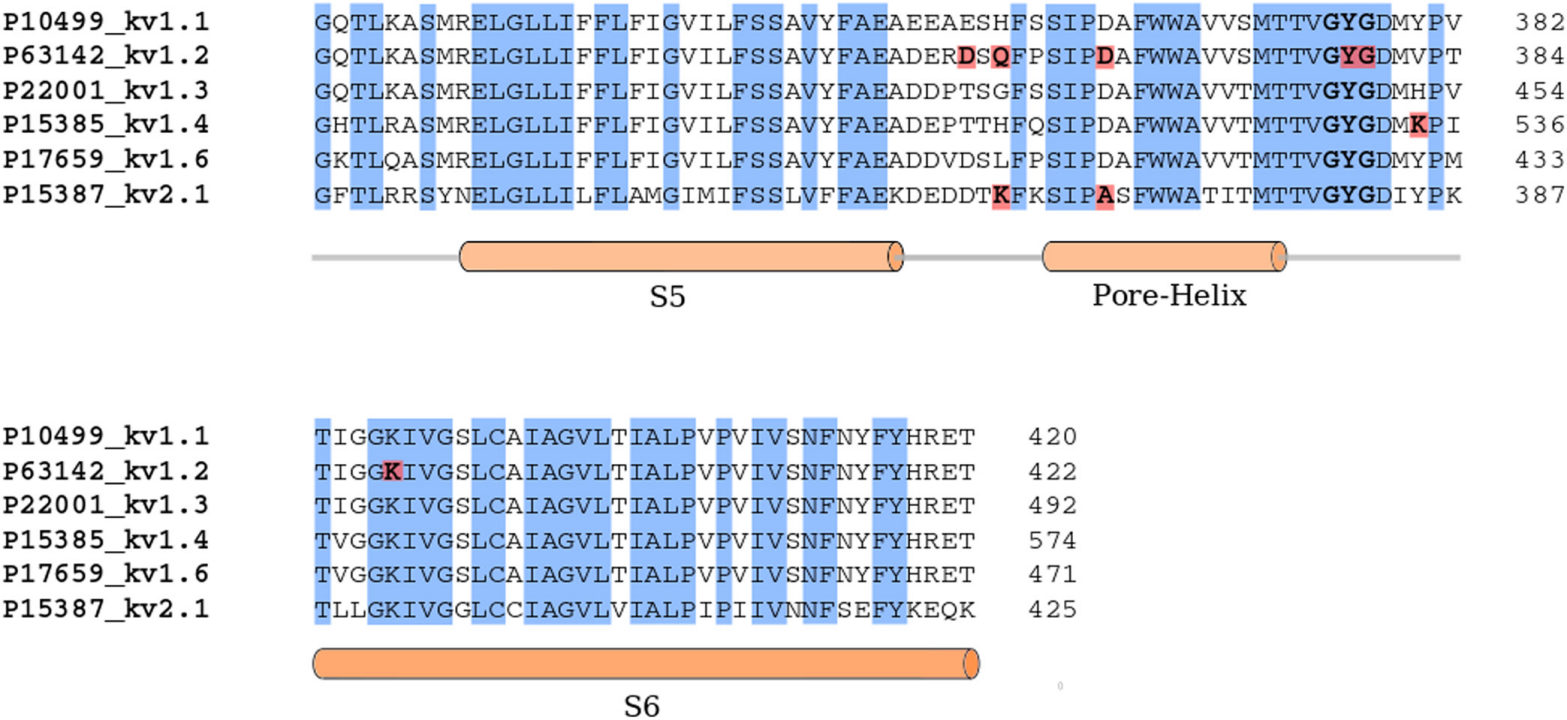
A multiple sequence alignment of the pore domain segments of the potassium channels Kv1.1, Kv1.2, Kv1.3, Kv1.4, Kv1.6 and Kv2.1 using 3D-Expresso program [43]. Conserved blocks are coloured in blue and the key residues for the interaction of Kbot21 with the channel are coloured in red boxes.

Electrophysiological studies have shown that Kbot21 is not active on the channel Kv2.1 and Kv1.4. These results are in agreement with our proposed model of interaction. Indeed for these two channels, the models suggest unfavourable interactions between the toxin and the channel residues. The presence of K360 in Kv2.1, instead of Q357 in Kv1.2 (Fig 6), could significantly destabilize the complex caused by the repulsive forces between the positively charged Kv2.1 residue and R6 and K28 of Kbot21. Similarly, charge repulsion may also occur between K531 of Kv1.4 and both R9 and R28 residues of Kbot21.

Further structure-function studies in which alanine scanning of the toxin combined with electrophysiological activities on different potassium channel subtypes (such as Kv, BK and SK channels) are required to verify if the residues suggested in our interaction model are indeed the key residues of the Kbot21 interaction with its target.

All these results show that small changes, whether in the channels or toxins could dramatically change their affinity for each other and data obtained in this work is helpful to reveal the diverse interactions between scorpion toxins and potassium channels and, therefore, can accelerate the molecular engineering of specific K_V_ channel inhibitors.

## Abbreviations

Bot: *Buthus occitanus tunetanus* scorpion
BotG50: Sephadex G-50 gel filtration toxic fraction of Bot venom
ELISA: enzyme-linked immunoabsorbent assay
i.c.v: intracerebroventricular
MALDI: Matrix-Assisted Laser Desorption Ionization
VGPC_S_: voltage gated potassium channels
